# Effects of Antifouling Biocides on Molecular and Biochemical Defense System in the Gill of the Pacific Oyster *Crassostrea gigas*

**DOI:** 10.1371/journal.pone.0168978

**Published:** 2016-12-22

**Authors:** Mi Seon Park, Young Dae Kim, Bo-Mi Kim, Youn-Jung Kim, Jang Kyun Kim, Jae-Sung Rhee

**Affiliations:** 1 Southeast Sea Fisheries Research Institute, National Institute of Fisheries Science, Tongyeong, South Korea; 2 Unit of Polar Genomics, Korea Polar Research Institute, Incheon, South Korea; 3 Department of Marine Science, College of Natural Sciences, Incheon National University, Incheon, South Korea; 4 Research Institute of Basic Sciences, Incheon National University, Incheon, South Korea; 5 Institute of Green Environmental Research Center, 169, Gaetbeol-ro, Yeonsugu, Incheon, South Korea; Institute of Oceanology, Chinese Academy of Sciences, CHINA

## Abstract

Antifouling biocides such as organotin compounds and their alternatives are potent toxicants in marine ecosystems. In this study, we employed several molecular and biochemical response systems of the Pacific oyster *Crassostrea gigas* to understand a potential mode of action of antifouling biocides (i.e. tributyltin (TBT), diuron and irgarol) after exposure to different concentrations (0.01, 0.1, and 1 μg L^-1^) for 96 h. As a result, all the three antifouling biocides strongly induced the antioxidant defense system. TBT reduced both enzymatic activity and mRNA expression of Na^+^/K^+^-ATPase and acetylcholinesterase (AChE). Lower levels of both Na^+^/K^+^-ATPase activity and *AChE* mRNA expression were observed in the diuron-exposed oysters compared to the control, while the irgarol treatment reduced only the transcriptional expression of *AChE* gene. We also analyzed transcript profile of heat shock protein (*Hsp*) superfamily in same experimental conditions. All antifouling biocides tested in this study significantly modulated mRNA expression of *Hsp* superfamily with strong induction of *Hsp70* family. Taken together, overall results indicate that representative organotin TBT and alternatives have potential hazardous effects on the gill of *C*. *gigas* within relatively short time period. Our results also suggest that analyzing a series of molecular and biochemical parameters can be a way of understanding and uncovering the mode of action of emerging antifouling biocides. In particular, it was revealed that Pacific oysters have different sensitivities depend on the antifouling biocides.

## Introduction

Biofouling represents the community of organisms that settle and grow on the external surfaces of submerged or semi-submerged objects. Biofouling is ubiquitous in the marine environments, causing, often time, detrimental problems for the shipping industry [1]. Tributyltin (TBT) was first used in freshwater systems to control mollusks and was introduced as an antifouling biocide in paints from the early 1960s [2]. The use of TBT and its exposure to the aquatic environment has been prominent [3, 4]. Extensive applications of TBT-based paints to vessels raise hazardous effects on many marine animals for several decades, particularly in mollusks (e.g. inhibition of spatfall, developmental abnormality, shell malformation, imposex, etc.) [5–7]. Organic booster biocides based on copper metal oxides and organic biocides were subsequently developed and introduced in antifouling products after the use of TBT globally was banned via legislation [8]. Most commonly used antifouling biocides as alternatives to TBT include chlorothalonil, dichlofluanid, DCOIT (4,5-dichloro-2-n-octyl-4-isothiazolin-3-one, Sea-nine 211^®^), diuron (1-(3,4-dichlorophenyl)-3,3-dimethylurea), Irgarol 1051 (2-(tert-butylamino)-4-(cyclopropylamino)-6-(methylthio)-s-triazine), TCMS pyridine (2,3,3,6-tetrachloro-4-methylsulfonyl pyridine), zinc pyrithione and Zineb [8]. Even tin-free alternatives can induce damages to marine ecosystems, and their potential persistence may also have diverse sublethal effects to marine animals on aspects from a molecular level to individual physiology and reproduction, or even a population level. Despite these concerns, the mode of action of these antifouling agents is virtually unknown in marine animals, and it is still difficult to assess resulting effects of alternatives on marine ecosystems.

Oysters are economically important as a major aquaculture species worldwide and play an important role in bridging the knowledge gap as a model invertebrate in estuarine and intertidal regions due to their global distribution. Because filter-feeders ingest suspended nutrients and food particles from water, they are likely to be impacted by diverse immune challenges and a wide range of anthropogenic influences. Since oysters accumulate metals and organic/inorganic compounds in tissue [9, 10], their biochemical and physiological parameters have been widely used as biomarkers of a marine environmental risk assessment [11]. Recently, the Pacific oyster *Crassostrea gigas* was highlighted as a reliable mollusk model for genome resource-based ecotoxicological and environmental research due to the public availability of reference genome information [12].

The increased use of antifouling biocides demands better understanding of molecular and biochemical mechanisms for sensitivity/toxicity, accumulation, detoxification, metabolism, and adaptation of aquatic invertebrates. Previous studies with the most commonly used antifouling agent, TBT show strong toxicity in relation to stress response. Basically, major toxicity of TBT derives from inhibition of oxidative phosphorylation in mitochondria, which alternate the ion transport across lipid membrane and inhibit the cytochrome P450 activity [13–15]. The released TBT readily bioaccumulates in lipid-rich tissues of aquatic organisms due to its low water solubility and lipophilicity. For example, the TBT concentration of up to 5.4 μg g^-1^ has been recorded in the Mediterranean mussel *Mytilus galloprovincialis* [16]. The ambient TBT concentration (≈ 1 ng L^-1^) in seawater can induce a masculinization of gonochoristic female mollusks through disruption of the hormone balance between oestrogens and androgens as in terms of imposex or intersex [17]. Another sublethal effect of TBT on mollusks is an abnormal calcification and subsequent distortion of shell via inhibition of the crystallization of calcium carbonate and interference of a shell component (organic matter (gel)) required for calcium deposition [5]. Diuron inhibits the Hill reaction in photosynthesis by binding to the photosystem II (PSII) complex in thylakoid membranes [3, 8]. Irgarol mainly functions by inhibiting electron transport in PSII [1, 8]. However, very few studies have conducted with the alternatives (i.e. diuron and irgarol) in analyzing response of molecular and biochemical metabolism in aquatic invertebrates. In *C*. *gigas*, the effects of diuron have continuously been reported from molecular to physiological level such as genotoxicity, embryotoxicity, spematotoxicity and immunity [18–24].

The overall objective of the present study is to analyze molecular and biochemical parameters of the Pacific oyster *C*. *gigas* upon exposure to antifouling biocides. In mollusks, the gill is the first organ exposed to water and diverse environmental factors including aquatic pollutants. We investigated the effects of three antifouling agents (i.e. TBT, diuron, and irgarol) on the lipid peroxidation, antioxidant defense system and heat shock protein (*Hsp*) expression in the gill tissues. Furthermore, we tested potential effects of antifouling biocides on both enzymatic activity and mRNA expression of Na^+^/K^+^-ATPase and acetylcholinesterase (AChE) to estimate whether these compounds have potential adverse effects on homeostasis of ionic/osmotic balance and cholinergic status. Our results will provide critical information to better understand the mode of action of emerging antifouling agents on the biochemical and physiological metabolism in Pacific oysters.

## Materials and Methods

### Culture and chemical exposure

The Pacific oyster *C*. *gigas* used in this study was collected from an Integrated Multi-Trophic Aquaculture (IMTA) system of Southeast Sea Fisheries Research Institute (National Institute of Fisheries Science; NIFS) in Tongyeong, Gyungnam, South Korea. This IMTA site is leased to Southeast Sea Fisheries Research Institute of National Institute of Fisheries Science (NIFS), Korea. No permission was required for sample collections. The pacific oyster is neither endangered nor protected species.

Adult oysters (≈ 103.8 ± 9.7 mm in length) were reared in filtered seawater with aeration at the automated culture system of Southeast Sea Fisheries Research Institute. The environmental conditions were maintained at 30 practical salinity unit (psu), 20°C and 16 h: 8 h L:D photoperiod. All the experiments using oysters were approved by the animal care and use committee of NIFS.

TBT, diuron and irgarol were purchased from Sigma (Sigma-Aldrich, Inc., St. Louis, MO, USA; purity > 99%), and their stock solutions (100 mg L^-1^) were prepared in dimethyl sulfoxide (DMSO; Sigma-Aldrich, Inc.). In our preliminary experiment, no significant mortality of oysters was observed up to 10 μg L^-1^ exposures of all three antifouling biocides for 96 h, while several susceptible oysters (e.g. oyster mortality, no response to external stimulus, etc.) were observed in the 25 μg L^-1^ TBT-exposed group. Although there was no significant detrimental effect in diuron- and irgarol-exposed oysters up to 50 μg L^-1^, we decided to analyze biochemical and physiological parameters within an environmentally relevant concentration range below 1 μg L^-1^ for all treatments (i.e. 0.01, 0.1, and 1 μg L^-1^). The ambient concentrations of biocides reported were 0.001–2.8 μg L^-1^ (TBT), 0.025–6.7 μg L^-1^ (diuron) and 0.006–1.4 μg L^-1^ (irgarol), respectively (Dafforn et al., 2011). Thus, all results presented in this study were considered as being sub-lethal effects within relatively short time period (96 h).

For the experiment, only healthy oysters were selected by observing their physiological responses (i.e., response of adductor muscle), as weak and/or dead oysters are occasionally observed during the acclimation. Adult oysters were exposed to different concentrations (i.e. 0.01, 0.1 and 1 μg L^-1^; nine oysters per seawater control, DMSO as a carrier solvent and each concentration, respectively) of antifouling biocides in seawater at 20°C for 96 h. In each exposed group, three oysters were collected and dissected for gill tissue preparation. The final maximum concentrations of DMSO in the treatment groups were maintained at less than 0.01%. In addition to seawater control, 0.01% DMSO was treated to validate solvent effect. During the experiment, half of the filtered seawater with an addition of equivalent concentration of each chemical was changed every 24 h. No diet was supplied during the experimental period due to adsorption potential or detoxification capacity of diet materials. Gill tissues from each oyster were analysed individually.

### Malondialdehyde (MDA) analysis

After dissection, gill tissues from three oysters were pooled as one replicate. Three replicates (totally nine oysters dissected for each group; seawater control, DMSO treatment and exposed groups) was independently analyzed. Approximately 62 ± 2.05 mg (n = 20) of gill tissues was used for each oyster. We followed a previously reported MDA analysis assay for *C*. *gigas* gill tissues [25]. Tissues were homogenized in 5 volumes of buffer (20 mM Tris, 150 mM NaCl, 10 mM β-mercaptoethanol, 20 μM leupeptin, 2 μM aprotinin and 100 μM benzamidine). After centrifugation for 30 min at 30,000 g (4°C), supernatants were heat-denatured for 15 min at 75°C. Thiobarbituric acid reactives (TBARs) were measured at 535 nm with a Thermo Varioskan Flash spectrophotometer (Thermo Fisher Scientific, Tewksbury, MA, USA) using malonaldehyde bis (tetrametoxypropan, Sigma-Aldrich, Inc.) as standard. The concentration of lipid peroxidation compounds was expressed as nM of MDA per gram of gill tissue.

### Enzymatic activity of antioxidant defense system

As we described, three pooled gill tissues from nine oysters per each group were independently analyzed as three replicate. Approximately 59 ± 1.23 mg of gill tissue was used for each replicate. All tissues were immediately washed or homogenized in different buffers as follows.

After 96 h of incubation at different antifouling biocide conditions, total glutathione (GSH) content and several enzyme activities (glutathione *S*-transferase, GST; glutathione peroxidase, GPx; glutathione reductase, GR; superoxide dismutase, SOD; catalase, CAT) were measured according to our previous study with slight modifications (e.g. buffer volume, employed basic instruments) [26–29]. GSH concentration was determined by an enzymatic method with the Glutathione Assay Kit (Catalog No. CS0260; Sigma-Aldrich, Inc.). After exposure to each chemical condition for 96 h, the gill tissue was washed in 0.9% NaCl. The rinsed samples were homogenized in trichloroacetic acid at a ratio of 1:20 (w/v) with a Teflon homogenizer. Each homogenate was centrifuged at 3,000 *g* for 10 min at 4°C. The upper aqueous layer was collected for the GSH content assay according to the manufacturer’s protocol. The GSH content was measured at an absorbance of 420 nm with a spectrophotometer, and the standard curves were generated with GSH equivalents (0, 150, and 350 μM).

The total GST activity was measured as described in our previous studies [26, 28]. After exposure to each chemical for 96 h, the gill tissue was homogenized in cold buffer [0.25 M sucrose, 10 mM Tris, 1 mM ethylenediaminetetraacedtic aced (EDTA), 0.2 mM dithiothreitol (DTT), and 0.1 mM phenylmethylsulfonyl fluoride (PMSF), pH 7.4] at a ratio of 1:4 (w/v) with a Teflon homogenizer. Each homogenate was centrifuged at 10,000 *g* for 10 min at 4°C. The cytosolic fraction containing the enzyme was collected for enzymatic assay with 1-chloro-2,4-dinitrobenzene (CDNB) as a substrate. The enzymatic assay monitored the conjugation of CDNB and GSH at 340 nm at 25°C. Total proteins were determined with the Bradford method [30]. Enzymatic activities were normalized by total protein and represented as a percentage of the control.

The GPx and GR activities were measured by enzymatic methods using Glutathione Peroxidase Cellular Activity Assay Kit (Catalog No. CGP1; Sigma-Aldrich, Inc.) and Glutathione Reductase Assay Kit (Catalog No. GRSA; Sigma-Aldrich, Inc.), respectively. After exposure to each chemical for 96 h, the gill tissue was homogenized in cold buffer (50 mM Tris-Cl, 5 mM EDTA, and 1 mM 2-mercaptoethanol, pH 7.5) at a ratio of 1:4 (w/v) with a Teflon homogenizer. Each homogenate was centrifuged at 10,000 *g* for 10 min at 4°C. The upper aqueous layer containing the enzyme was collected for the enzymatic assay according to the manufacturer’s protocol. The GPx and GR activities were then measured at an absorbance of 340 nm at 25°C.

The SOD and CAT activities were measured with an enzymatic method using SOD Assay Kit (Catalog No. 19160; Sigma-Aldrich Chemie, Switzerland) and Catalase Assay Kit (Catalog No. CAT100; Sigma-Aldrich, Inc.), respectively. After exposure to environmental biocides for 24 h, the gill tissues were homogenized in ice-cold buffer (0.25M sucrose, 0.5% triton X-100, pH 7.5) at a ratio of 1 to 4 (w/v) using a Teflon homogenizer. The homogenate was centrifuged at 3,000 g for 30 min at 4°C. The upper aqueous layer containing the enzyme was collected for the enzymatic assay according to the manufacturer’s protocol. The total SOD activity and CAT activity were then measured at an absorbance of 440 nm or 520 nm at 25°C, respectively.

The Na^+^/K^+^-ATPase activity was measured using the method of McCormick [31] as used for detection in waterflea [32] and mussel [33]. Briefly, the method calculates the difference in the amount of adenosine diphosphate (ADP) produced by the samples between two reaction mixtures, a control reaction (no inhibitor added) and in an inhibition reaction [33]. After exposure to antifouling biocides, the gill tissue was homogenized in ice-cold buffer (150 mM sucrose, 10 mM ethylenediaminetetraacetic acid, 50 mM imidazole, and 11.5 mM sodium deoxycholate), at a ratio of 1 to 5 (w/v) using a Teflon homogenizer. The homogenate was centrifuged at 5,000 g for 1 min at 4°C. The upper aqueous layer containing the enzyme was collected for the Na^+^/K^+^-ATPase enzymatic assay. Difference between the production of inorganic phosphate in the buffer and the production of phosphate in buffer without K was quantified. Ouabain (Sigma-Aldrich, Inc.) was used as an inhibitor of the reaction. The reaction was analyzed at an absorbance of 340 nm at 20°C. Enzyme activity was finally expressed as μmoles ADP/mg protein/h.

Based on method established by Ellman et al. [34], AChE enzymatic activity was measured according to our previous study with slight modifications (e.g. buffer volume, employed basic instruments) [35, 36]. Acetylthiocholine iodide (ATCh) and 5,5′-dithiobis (2-nitrobenzoic acid) (DTNB) were purchased from Sigma (Sigma-Aldrich, Inc.). The reaction mixture was prepared in 100 mM of potassium phosphate buffer (pH 7.4). Each exposed gill tissue was used for each concentration. After exposure to antifouling biocides, the gill tissue was homogenized in ice-cold phosphate buffer (0.1 M, pH 8.0) at a ratio of 1 to 5 (w/v) using a Teflon homogenizer. The homogenate was centrifuged at 3,000 g for 30 min at 4°C. The upper aqueous layer containing the enzyme was collected for the AChE enzymatic assay. Then, 100 μL of the supernatant was added to 1.3 mL of the phosphate buffer (0.1 M, pH 8.0) in a 3 mL cuvette, and 50 μL of DTNB (0.01 M) and 10 μL of ATCh (0.075 M) were added as a substrate. The total AChE enzymatic activity was measured using ATCh, a blank without acetylthiocholine and a blank without sample for 5 min at an absorbance of 412 nm at 25°C. The enzymatic activity was normalized to total protein in supernatant and was represented as a percentage of the control. AChE activity was calculated in nmoles of hydrolyzed acetylcholine chloride/min/mg protein (extinction coefficient ε_412_ = 13,600 M^-1^ cm^-1^).

### Total RNA extraction and single-strand cDNA synthesis

Each gill tissue (≈ 47 ± 1.57 mg) was homogenized in 3 volumes of TRI Reagent^®^ (Sigma-Aldrich, Inc.) with a Teflon homogenizer. Total RNA was extracted according to manufacturers’ instructions and stored at -80°C until use. DNA digestion was performed using RNase-Free DNase Set (QIAGEN, Valencia, CA, USA). Total RNA was quantified by absorption of light at 230, 260, and 280 nm (A230/260, A260/280) using a NanoDrop^®^ 2000 Spectrophotometer (Thermo Scientific, Wilmington, DE, USA). To check genomic DNA contamination, total RNA was loaded in a 1% agarose gel which contained ethidium bromide (EtBr) and visualized it on a UV transilluminator (Wealtec Corp., NV, USA). Subsequently, total RNA was loaded in a 1% formaldehyde/agarose gel with EtBr staining to verify for total RNA quality, and checked the 18/28S ribosomal RNAs integrity. After RNA quality was determined, single-strand cDNA was synthesized from 2 μg of total RNA from each sample using oligo (dT)_20_ primer for reverse transcription in 20 μl reactions using QuantiTect Reverse Transcription Kit (QIAGEN, Valencia, CA, USA). Using RT Primer Mix (QIAGEN), reverse transcription was performed in T100™ Thermal Cycler (Bio-Rad, Hemel Hempstead, UK) according to manufacturers' instructions under the following condition: Pre-incubation 42°C for 2 min; incubation with Quantiscript Reverse Transcriptase (QIAGEN) for 15 min; Inactivation 95°C for 3 min. A no template control (NTC) reaction was conducted in order to validate DNA contamination in buffers/solutions.

### Real-time reverse transcriptase-polymerase chain reaction (real-time RT-PCR)

Transcriptional responses of *C*. *gigas* heat shock protein (*Hsp*) superfamily, Na^+^/K^+^-ATPase α subunit, and *AChE* were analyzed using real-time reverse transcriptase-polymerase chain reaction (RT-PCR) based on the Minimum Information for Publication of Quantitative Real-Time PCR Experiments (MIQE) guidelines [37]. Specific gene information and details of confirmed primer sets of *C*. *gigas* antioxidant defence system (i.e. GST omega, *GSTO*; GST pi, *GSTP*; GST sigma, *GSTS*; *GPx*; *GR*; Copper/Zinc SOD, *CuZnSOD*; Manganese SOD, *MnSOD*; and Catalase, *CAT*), heat shock protein (*Hsp*) superfamily and Na^+^/K^+^-ATPase α subunit were retrieved from previous studies [38–41] and were appended (S1 Table). Of 21 *hsps*, polymerase chain reactions of three *hps70* genes (i.e. *hsp70-15492*, *hsp70-27129*, and *hsp70-22078*) were not efficiently amplified in our experimental conditions, and thus the data was excluded for analysis. Real-time RT-PCR was conducted using the SYBR^®^ Green Master Mix (Bio-Rad). Reactions were run at a final volume of 25μl consisting of the following master mix: 12.5μl of SYBR Green buffer, 1μl each of forward and reverse primers (250 nM), 9.5μl nuclease-free water, and 1μl cDNA (total RNA equivalent). Amplification and detection of SYBR Green-labelled amplicons was performed with optimized conditions using the CFX96 real-time PCR system (Bio-Rad, Hercules, CA, USA) under the following conditions: 95°C/5 min; 40 cycles of 95°C/20 s, 55°C/30 s, and 72°C/40 s. All real-time RT-PCR experiments were carried out in unskirted low 96-well clear plates (Bio-Rad). To confirm the amplification of specific products, cycles were continued to determine the melting curve under the following conditions: 95°C/1 min, 55°C/1 min, and 80 cycles of 55°C/10 s with a 0.5°C increase per cycle. To set an appropriate reference gene for the real-time RT-PCR as a preliminary experiment, reliability of 7 reference candidates was validated using intra- and inter-laboratory validation procedures in a multiplex PCR condition (*18S rRNA*; *28S rRNA*; glyceraldehyde 3-phosphate dehydrogenase, *GAPDH*; *β-actin*; ribosomal protein L7, *RL7*; ribosomal protein S18, *RPS18*; elongation factor 1 α, *EF1α*) at same experimental conditions. Specific gene information and details of primer sets of *C*. *gigas* housekeeping genes were retrieved from Zhu et al. [41]. As a result, two reference genes, *EF1α* (average stability: 0.226 for TBT, 0.213 for diuron, 0.243 for irgarol, respectively) and *GAPDH* (average stability: 0.231 for TBT, 0.265 for diuron, 0.259 for irgarol, respectively) genes showed the most stable expression pattern in most samples. Thus, data from triplicate experiments were expressed relative to expression of the internal control *EF1α* gene. Each transcriptional level was determined by the 2^-ΔΔC^t method [42] (Livak and Schmittgen, 2001). Heat map and hierarchical clustering analysis were conducted to represent the transcript profile using MeV software (ver. 7.4; Dana-Farber Cancer Institute, Boston, MA, USA).

### Statistical analysis

The SPSS ver. 17.0 (SPSS Inc., Chicago IL, USA) software package was used for statistical analysis. Data are expressed as means ± standard deviation (S.D.). Significant differences between the observations of control and exposed groups were analyzed using one-way comparison ANOVA followed by Tukey’s test. Any difference showing *P* < 0.05 was considered significant.

## Results

### MDA content

**Fig 1:**
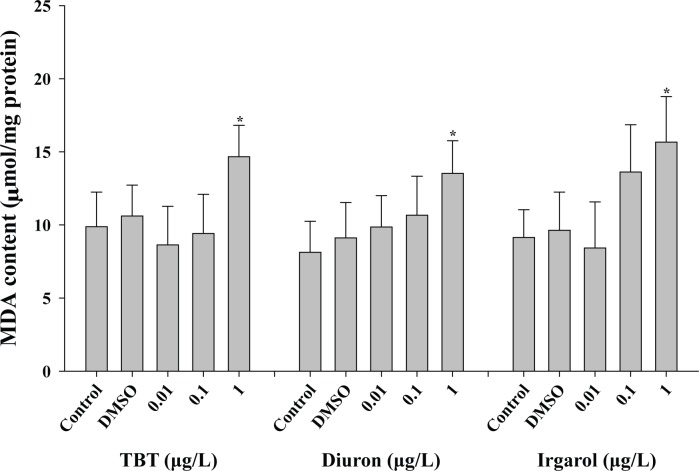
Effects of three antifouling biocides, TBT, diuron, and irgarol on MDA content in the gill of *Crassostrea gigas*. Data are presented as the mean ± standard deviation (S.D.). Significant difference compared with control value is indicated by an asterisk (*) on the data bar (*p* < 0.05).

Significant increases of MDA content were observed in the gill tissues upon 1 μg L^-1^ (14.7 ± 2.15 μM mg^-1^) of TBT (*p* < 0.05) compared to the control value (9.9 ± 2.36 μM mg^-1^) (Fig 1). The highest MDA levels were observed at 1 μg L^-1^ diuron (13.5 ± 2.24 μM mg^-1^) and irgarol (15.7 ± 3.11 μM mg^-1^) (*p* < 0.05) compared to the control levels (8.13 ± 2.11 μM mg^-1^ for diuron; 9.14 ± 1.90 μM mg^-1^ for irgarol, respectively). There is no significant MDA change when exposed to the DMSO treatment (0.01%).

### Enzymatic activity and transcriptional response of antioxidant defense system

**Fig 2:**
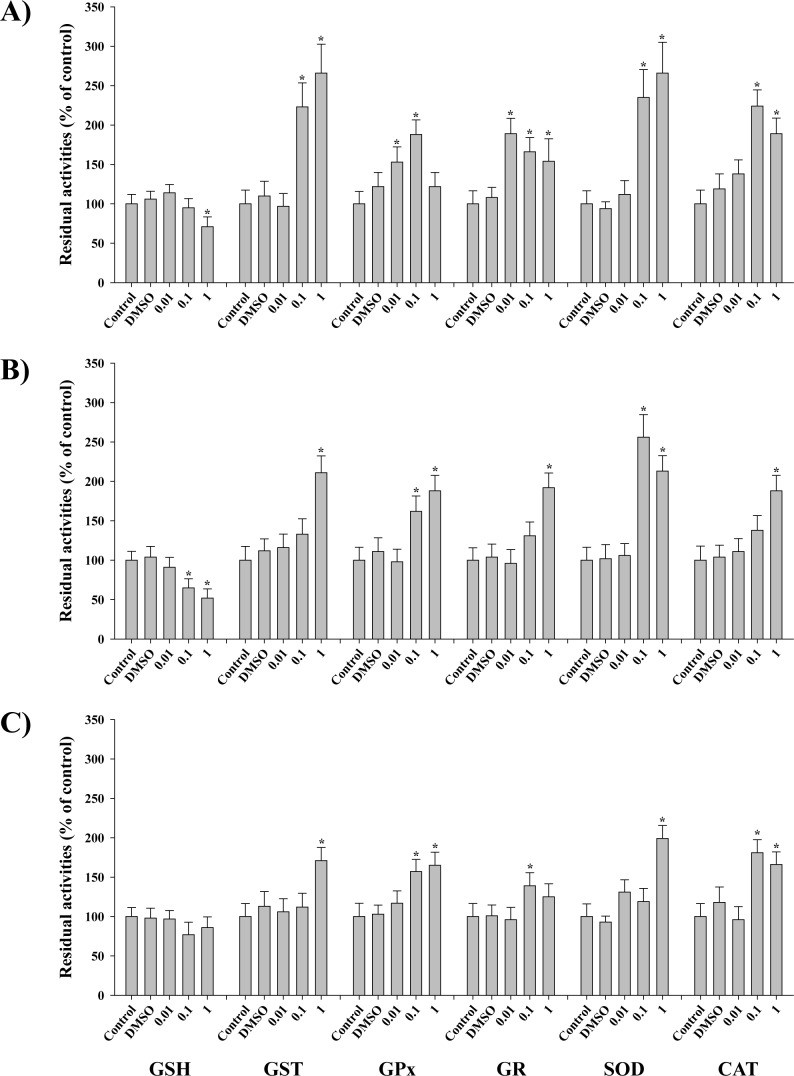
Effects of three antifouling biocides, TBT, diuron, and irgarol on antioxidant defense system in the gill of *Crassostrea gigas*. A) Results of TBT-exposed oysters. B) Results of diuron-exposed oysters. C) Results of irgarol-exposed oysters. The remaining activities were recorded as percentages relative to the control. Data are presented as the mean ± standard deviation (S.D.). Significant difference compared with control value is indicated by an asterisk (*) on the data bar (*p* < 0.05).

**Fig 3:**
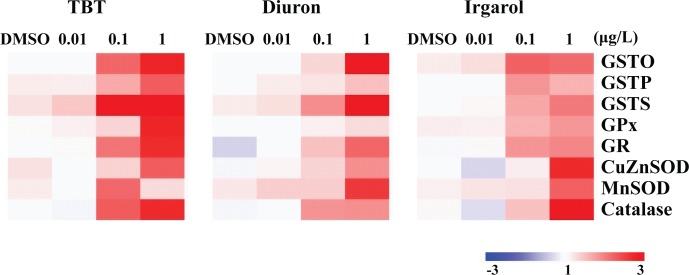
Transcriptional profiles of *Crassostrea gigas* antioxidant defense system in response to different concentrations of TBT, diuron, and irgarol. Heat map analysis for effects of TBT, diuron, and irgarol on transcriptional expressions of *Hsp* superfamily.

GSH was depleted at 1 μg L^-1^ TBT (71%) (*p* < 0.05; Fig 2A). Absolute control values for each chemical were as follows; TBT: GST 12.66 ± 1.28 mU mg^-1^, GPx 4.58 ± 0.51 mU mg^-1^, GR 2.85 ± 0.21 mU mg^-1^, SOD 14.52 ± 1.11 U mg^-1^, CAT 93.52 ± 8.95 U mg^-1^; Diuron: GST 13.62 ± 1.31 mU mg^-1^, GPx 5.14 ± 0.67 mU mg^-1^, GR 3.1 ± 0.19 mU mg^-1^, SOD 15.34 ± 1.08 U mg^-1^, CAT 89.21 ± 7.93 U mg^-1^; Irgarol: GST 13.1 ± 1.17 mU mg^-1^, GPx 4.79 ± 0.42 mU mg^-1^, GR 2.66 ± 0.24 mU mg^-1^, SOD 14.29 ± 1.31 U mg^-1^, and CAT 101.11 ± 10.42 U mg^-1^, respectively. GST, SOD, and CAT activities were significantly increased at 0.1 (223% for GST; 235% for SOD; and 224% for CAT, respectively) and 1 μg L^-1^ (266% for GST; 266% for SOD; and 189% for CAT, respectively) of TBT (*p* < 0.05), while significant increases of GPx and GR were observed when exposed to 0.01 (153% for GPx; and 189% for GR, respectively), 0.1 (188% for GPx; and 166% for GR, respectively) or 1 μg L^-1^ (154% for GR) of TBT (*p* < 0.05). In the case of diuron, the exposure to 0.1 (65%) and 1 μg L^-1^ (52%) for 96 h caused a significantly depleted total GSH content (*p* < 0.05; Fig 2B). GST, GR, and CAT activities showed a similar pattern. The highest GST, GR, and CAT activities were observed at 1 μg L^-1^ diuron (211% for GST; 192% for GR; and 188% for CAT, respectively) (*p* < 0.05). GPx and SOD activities were elevated at 0.1 (162% for GPx; 256% for SOD) and 1 μg L^-1^ (188% for GPx; and 213% for SOD, respectively) of diuron (*p* < 0.05). We also observed significant inductions of antioxidant enzyme activities at 0.1 (171% for GST; 157% for GPx; 139% for GR; and 181% for CAT, respectively) or 1 μg L^-1^ (165% for GPx; 199% for SOD; and 166% for CAT, respectively) of irgarol (*p* < 0.05) although significant modulation was not observed in GSH (Fig 2C). Overall enzymatic activity of antioxidant defense system appeared to be lower in the irgarol-exposed group compared to TBT- or diuron-exposed oysters. Similarly, overall transcriptional profiles of antioxidant defense system were increased at the highest concentration (1 μg L^-1^) (Fig 3). In the TBT-exposed oysters, *GSTO*, *GSTS* and *CAT* were significantly expressed over two-fold in both 0.1 and 1 μg L^-1^ of TBT (*p* < 0.05). Significant up-regulations of *GSTP*, *GPx*, *GR* and *CuZnSOD* were observed in 1 μg L^-1^ of TBT (*P* < 0.05, over two-fold). *GSTO*, *GSTS* and *MnSOD* showed significant increase in 1 μg L^-1^ of diuron (*P* < 0.05, over two-fold). Irgarol exposure increased mRNA expressions of *GSTO*, *CuZnSOD*, *MnSOD* and *CAT* genes at 1 μg L^-1^ (*P* < 0.05, over two-fold).

### Enzymatic activity and transcriptional response of Na^+^/K^+^-ATPase

**Fig 4:**
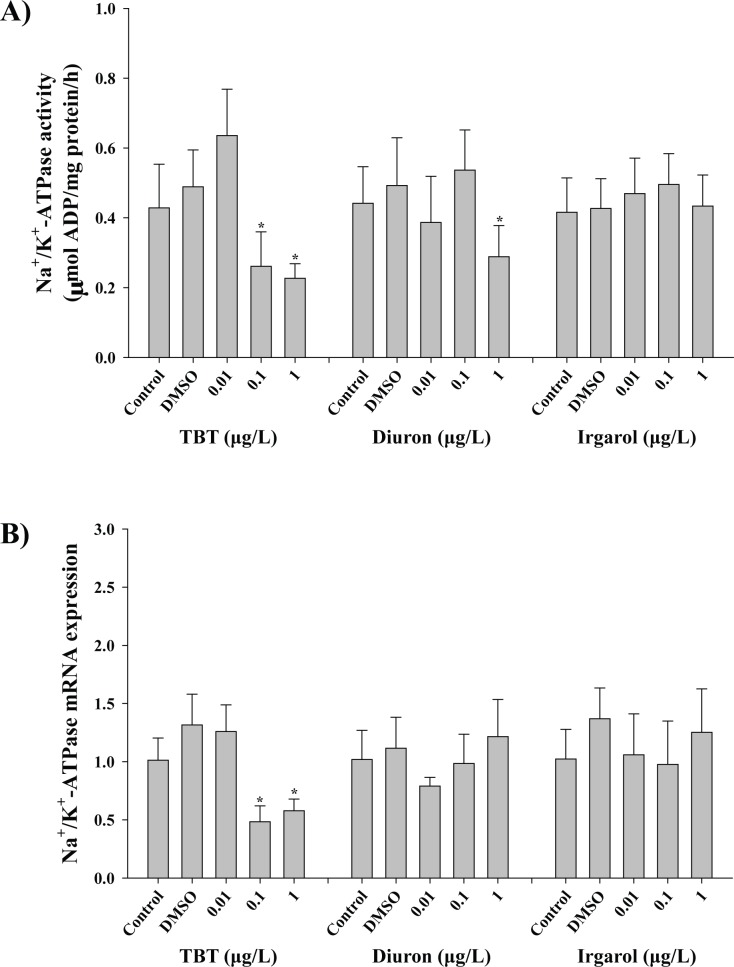
Effects of three antifouling biocides, TBT, diuron, and irgarol on Na^+^/K^+^-ATPase in the gill of *Crassostrea gigas*. A) Effects of TBT, diuron, and irgarol on enzymatic activity of Na^+^/K^+^-ATPase. B) Effects of TBT, diuron, and irgarol on mRNA expression of Na^+^/K^+^-ATPase α subunit. Data are presented as the mean ± standard deviation (S.D.). Significant difference compared with control value is indicated by an asterisk (*) on the data bar (*p* < 0.05).

When the oysters were exposed to different concentrations of TBT, the significant lower enzymatic activity of Na^+^/K^+^-ATPase was observed at 0.1 (0.26 ± 0.10 μM mg^-1^) and 1 μg L^-1^ (0.22 ± 0.04 μM mg^-1^) of TBT (*p* < 0.05) compared to the control (0.42 ± 0.13 μM mg^-1^) (Fig 4A). The lowered activity of Na^+^/K^+^-ATPase enzyme was also observed at 1 μg L^-1^ diuron (0.29 ± 0.09 μM mg^-1^) (*p* < 0.05). Transcriptional expressions of Na^+^/K^+^-ATPase α subunit were further checked following different concentrations of three antifouling biocides (Fig 4B). Similarly, a lower level of mRNA expression was measured at 0.1 (0.48 ± 0.14) and 1 μg L^-1^ (0.58 ± 0.10) of TBT (*p* < 0.05) compared to the control (1.01 ± 0.19), while no significant change was observed in both diuron- and irgarol-exposed oysters (*p* > 0.05).

### Enzymatic activity and transcriptional response of acetylcholinesterase

**Fig 5:**
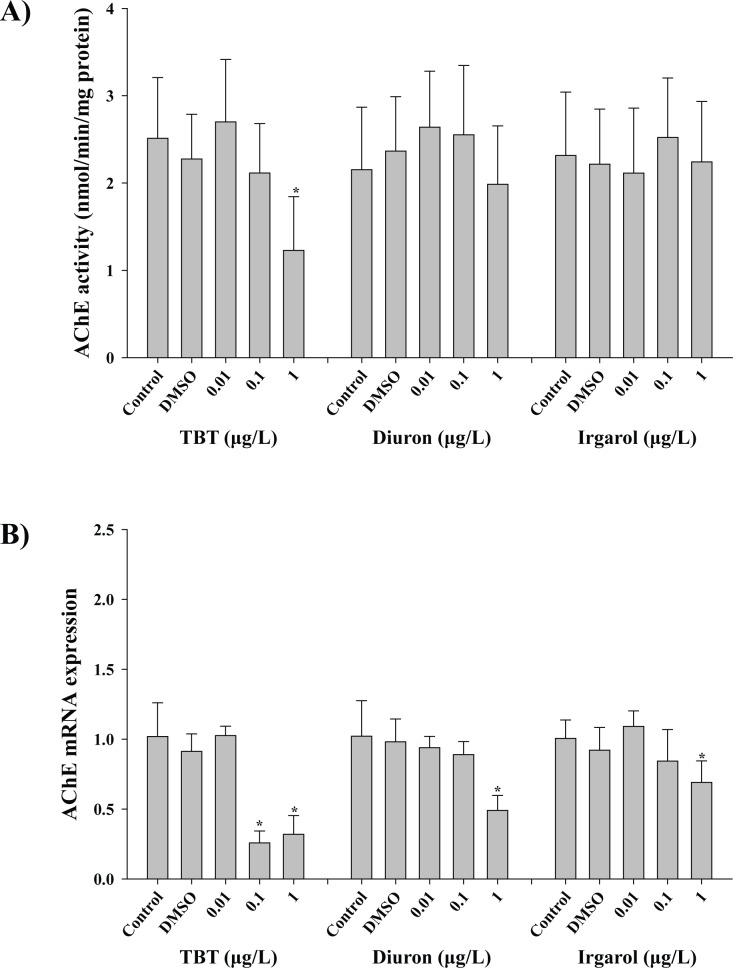
Effects of three antifouling biocides, TBT, diuron, and irgarol on acetylcholinesterase (AChE) in the gill of *Crassostrea gigas*. A) Effects of TBT, diuron, and irgarol on enzymatic activity of AChE. B) Effects of TBT, diuron, and irgarol on mRNA expression of *AChE* gene. Data are presented as the mean ± standard deviation (S.D.). Significant difference compared with control value is indicated by an asterisk (*) on the data bar (*p* < 0.05).

A significant lower level of the enzyme activity of AChE was observed at 1 μg L^-1^ (1.23 ± 0.619 nM mg^-1^) of TBT treatment (*p* < 0.05) compared to the control (2.51 ± 0.69 nM mg^-1^), while diuron and irgarol did not induce any significant effect at 96 h (*p* > 0.05) (Fig 5A). The *AChE* mRNA expression was decreased at 0.1 (0.26 ± 0.09) and 1 μg L^-1^ (0.32 ± 0.13) of TBT and 1 μg L^-1^ of diuron (0.49 ± 0.11) and irgarol (0.69 ± 0.15) (*p* < 0.05) (Fig 5B).

### Transcriptional profiles of heat shock protein (*Hsp*) superfamily

**Fig 6:**
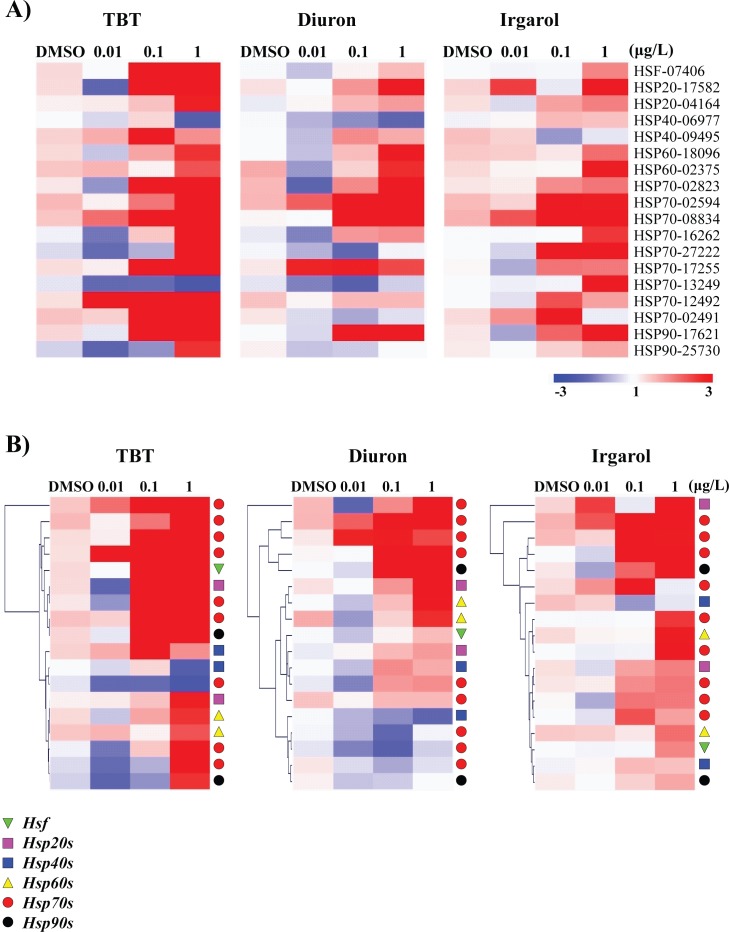
Transcriptional profiles of *Crassostrea gigas* heat shock protein (*Hsp*) superfamily in response to different concentrations of TBT, diuron, and irgarol. A) Heat map analysis for effects of TBT, diuron, and irgarol on transcriptional expressions of *Hsp* superfamily. B) Expression profiles are represented by a heat map with hierarchical clustering analysis.

To investigate transcriptional changes of heat shock protein (*Hsp*) superfamily after exposure to antifouling biocides, we measured mRNA expressions of one heat shock factor (*Hsf*) and seventeen *Hsps* gene that are retrieved from a previous study [41]. Analysis of the overall effect of antifouling biocides across the three concentrations relative to an unexposed group revealed altered expressions of *Hsp* genes at 96 h (Fig 6A). Most of *Hsp* genes (*P* < 0.05, over two-fold: *HSP20-17582*, *HSP60-18096*, *HSP70-02491*, *HSP70-02594*, *HSP70-02823*, *HSP70-08834*, *HSP70-12492*, *HSP70-16262*, *HSP70-17255*, *HSP70-27222*, and *HSP90-17621*, respectively) were strongly up-regulated at 1 μg L^-1^ TBT. mRNA expressions of several *Hsp70* isoforms were also induced at 1 μg L^-1^ of diuron (*P* < 0.05, over two-fold: *HSP70-02823*, *HSP70-02594*, *HSP70-08834*, and *HSP70-17255*, respectively) and irgarol (*P* < 0.05, over two-fold: *HSP70-02594*, *HSP70-08834*, *HSP70-27222*, and *HSP70-13249*, respectively) at 96 h. Apparent up-regulations of *Hsf* gene were only detected in the 0.1 and 1 μg L^-1^ of TBT-exposed groups (*P* < 0.05). One of *Hsp20* family (*Hsp20-17582*) or *Hsp90* family (*Hsp90-17621*) was constantly up-regulated at 1 μg L^-1^ of all three chemicals (*P* < 0.05, over two-fold). *Hsp40* (*Hsp40-06977*) and *Hsp70* (*Hsp-02594*; *Hsp-13249*) families were down-regulated at 1 μg L^-1^ of TBT or irgarol (*P* < 0.05). Hierarchical clustering analysis represented that the most acutely up-regulated *Hsp* genes belonged to *Hsp70* family as marked with red circles (Fig 6B).

## Discussion

To date, many non-enzymatic parameters and antioxidant enzymes have been identified from marine mollusks, and their potential roles as biomarkers upon stressful oxidative condition have been drastically investigated. Briefly, free radicals or pro-oxidant molecules can lead to oxidative stress with an induction of lipid peroxidation. MDA is one of byproducts of lipid peroxidation and thus is widely used as a strong biomarker for assessing oxidative stress. CAT and SOD are the first line of defense against oxidative stress. GR and GPx contribute cellular antioxidant protection and catalyze the reduction of H_2_O_2_ and lipid peroxides [43]. In oysters, the defense system also has primarily been studied as potential biomarkers of oxidative stress [44, 45], but information on stress effects of antifouling compounds is still limited. We observed significant increases of MDA contents. This result suggests that antifouling agents may increase of intracellular reactive oxygen species (ROS) and subsequently induce lipid peroxidation in the gill tissue. Overall response of antioxidant defense system with an increase of transcriptional profile suggests that the exposure to the antifouling biocides (e.g. tributyltin (TBT), diuron and irgarol) can be an oxidative stress inducer in *C*. *gigas*. Similar results of strong modulation in antioxidant defense system were found in other aquatic animals, such as marine algae, nematode, coral, and fish when they were exposed to TBT, diuron, or irgarol [46–50].

*In vitro* tests revealed that TBT and irgarol treatments induce apoptosis via increase of ROS and release of Ca^2+^ [51–53]. Diuron exposure (300 ng L^-1^ and 3 μg L^-1^ for 11 weeks) increased ROS level in the hemocyte of *C*. *gigas* [54]. Parental exposure to diuron resulted in oxidative DNA damage with an increase in 8-oxodGuo levels to germinal cells of *C*. *gigas* [23]. Although there is no evidence on the increase of intracellular ROS in the *C*. *gigas* gill tissue, the enzymatic and transcriptional inductions of antioxidant proteins may be primarily due to the result of antifouling biocides-triggered intracellular oxidative stress in *C*. *gigas*.

GSH participates in the antioxidant defense system to maintain the intracellular redox homeostasis. Elevated GSH level can increase the resistance capacity against oxidative stress, while GSH depletion can lead to susceptibility. In this study, the lower levels of GSH were observed in the TBT- and diuron-exposed *C*. *gigas*, but most enzymatic activities were elevated. Response of GSH and antioxidant defense system is controversial, as the system can be differentially modulated by tissues, chemical concentration and/or time-course in animals. Marbled rockfish (*Sebastiscus marmoratus*) exposed to different concentrations (1, 10, 100 ng L^-1^) of TBT showed significant depletions of total GSH content in 50 days and recovery period (7 days) [55]. However, increases or decreases of enzymatic activity of GPx and GST were differentially modulated by TBT concentrations or exposure/recovery periods. Although the GSH pattern should be monitored for time-course in *C*. *gigas*, the lower levels of GSH in the TBT- and diuron-exposed oysters can be explained as conjugation of GSH with the two antifouling agents. Basically, GSH depletion increases the rate of GSH synthesis through a negative feedback mechanism by the activity of γ-glutamylcysteine synthetase (γ-GCS) [56]. However, prolonged GSH depletion by antifouling agents could reduce the detoxification capacity and induce the vulnerability to the oxidative stress. Regarding transcriptional increase of antioxidant defense system in *C*. *gigas*, GSH depletion may partially support the previous important study that transcriptional expressions of antioxidant enzymes are induced by GSH depletion via the antioxidant response element (ARE) in the promoter region of the genes by binding of the nuclear factor-like 2 (Nrf2) transcription factor [57].

Na^+^/K^+^-ATPase is a ubiquitously expressed transmembrane protein complex acting as a key energy-consuming pump for maintaining ionic and osmotic balance in aquatic animals [58]. To expand our knowledge on the effects of antifouling biocides on ATP consumption and ionic metabolism, Na^+^/K^+^-ATPase enzyme activity and Na^+^/K^+^-ATPase α subunit transcript were further examined. The expressions of Na^+^/K^+^-ATPase enzyme activity and Na^+^/K^+^-ATPase α subunit transcript were down-regulated by TBT exposure, indicating potential toxicity of TBT. As described in ‘Materials and Methods,’ *C*. *gigas* showed higher sensitivity upon TBT exposure than diuron or irgarol at the preliminary toxicity test. Suppression of Na^+^/K^+^-ATPase by organotins has been studied in vitro model system, fish, and mammals. The induction of intracellular oxidative stress by organotins caused membrane depolarization and inhibition of neurotransmission [50, 59–62]. This result suggests that lowered Na^+^/K^+^-ATPase activity might be a potential reason for the high sensitivity in *C*. *gigas* when exposed to TBT, although the experiment on relationship between Na^+^/K^+^-ATPase activity and oyster mortality should be extended.

In addition to the result of TBT exposure, the pattern of enzymatic activity level was comparable to the level of its transcript and thus both enzyme activity and mRNA expression of Na^+^/K^+^-ATPase are a promising biomarker for TBT exposure based on the clear correlations. In the case of diuron, significant low enzyme activity was measured in the highest concentration (1 μg L^-1^) tested in this study without transcriptional modulation. Irgarol, however, did not show any significant effect on both parameters in the gill tissue of *C*. *gigas*. Although a wide range of concentration and exposure period should be conducted, diuron appears to have a potential to modulate the ionic homeostasis and osmotic balance in the gill tissues of *C*. *gigas*.

Regarding lowered levels of both AChE activity and its transcript by the TBT treatment, our results suggest the toxic cholinergic effect of TBT in *C*. *gigas*. Acetylcholine (ACh) is the primary neurotransmitter of sensory and nervous system, and found in almost all aquatic animals. In general, AChE, a ubiquitous class of serine hydrolases, has a crucial role in neurotransmitter release and synaptic plasticity by degradation of the ACh for the transmission of nervous signals in cholinergic synapses [63]. The inhibition of AChE activity delays the decrease of acetylcholine and interferes with the normal nerve fiber communication, resulting an adverse effect on physiology of individual organism and/or ecosystem, which eventually leads to significant mortality. Thus, TBT contaminant can induce impairment of the nervous system function in the gill tissues of *C*. *gigas*. Diuron and irgarol also significantly inhibited mRNA expression of *AChE* at 96 h, but no critical change was observed in its enzymatic activity. Previously, a significant correlation of *AChE* mRNA expression with its protein activity was observed in copepods and rotifers upon biocide or pharmaceutical exposures [35, 64]. Therefore, the transcriptional modulation of *AChE* can affect its activity in a time-course, but further study should be conducted to clarify employing different concentrations and exposure durations. TBT-triggered lowered enzyme activities of AChE with Na^+^/K^+^-ATPase may also induce a negatively synergistic role as neurotoxicity due to their basic roles in nerve synapse and ionic environment. In addition, the oxidative stress induced by antifouling biocides can be an indirect way to induce neurotoxicity as the intracellular oxidative stress contributes to the induction of neurodegenerative disorders [65]. Previous reports suggested that TBT exposure induced significant neurological damage with an induction of oxidative stress [50, 62].

Environmental pollutants can induce a series of molecular and systemic mechanisms for maintaining cellular and physiological homeostasis. Hsps, a ubiquitous type of stress proteins, are expressed by a wide variety of physiological and environmental stimuli for their predominant function being the folding and unfolding of proteins [66, 67]. In the present study, most *hsps*’ transcriptional levels were highly modulated in response to antifouling biocides. Overall, the most acutely up-regulated *Hsp* genes identified in *C*. *gigas* belonged to *Hsp70* family. In *Crassostrea* sp., *Hsp70* family has been considered as the central components of the cellular network of molecular chaperones and a strong inducible biomarker to assess pollution in the aquatic environment [11, 38, 68–71]. Based on whole *Hsp* profiles, TBT treatment seemed to be a stronger stressor than diuron or irgarol in the gill tissues of *C*. *gigas*. We suppose that even relatively low concentration of TBT could induce a strong cell stress in *C*. *gigas*. Our results also showed that *C*. *gigas* had relatively higher sensitivity to TBT exposure than diuron or irgarol. In fact, TBT contamination is known to be a strong stressor to induce *Hsps* in aquatic animals including mollusks [50, 72–76]. In addition, *C*. *gigas* represents different specificity and sensitivity against the types of biocide, exposure concentration and duration. For example, no induction of *Hsp70* was found when *C*. *gigas* was exposed to three different biocides with different doses (c.a. 5 μg L^-1^ carbofuran, 2 μg L^-1^ lindane, and 10 μg L^-1^ metolachlor for 30 days) suggesting a low-dose effect [77]. However, strong transcriptional modulations of *Hsps* were observed in the diuron-exposed *C*. *gigas* (two 7-day exposure pulses to 0.2–0.3 μg L^-1^ diuron) [24]. These results suggest that up-regulated *Hsp* transcripts generated by the antifouling biocide treatments confer cell protection which enables oysters to survive from the detrimental damage.

The antifouling biocides tested in this study induced oxidative stress. This result suggests that intracellular oxidative stress may induce *Hsp* expressions as an indirect effect of antifouling biocides. Imbalance between cellular redox state and detoxification capacity of oxygen metabolites leads to an accumulation of oxidized intracellular components such as DNA, lipids and proteins, resulting in the failure of normal cell function [78]. Hsps can strongly intervene to diminish oxidative stress-triggered damage via chaperonin function, sorting and selecting of aberrant proteins to proteasome for degradation, and other versatile roles [66]. In fact, a previous study suggested that Hsp70 modulates the activities of GPx and GR as Hsp70’s cytoprotective effects [79]. Thus, some up-regulated *Hsps* may be involved in the oxidative stress relevant cell damage and apoptotic mortality in *C*. *gigas*.

Higher concentration or accumulation of antifouling biocides interferes with molecular and cellular defense mechanisms via their persistency in aquatic environment, particularly in filter-feeding mollusks. Bioaccumulation of waterborne TBT in mollusks is of broad interest due to its endocrine disrupting effects such as imposex [17, 80]. Diuron is known to have moderate persistency in the aquatic ecosystems [4, 81], while irgarol and its toxic biotransformed products have relatively high persistency in water [4]. Further studies are needed to compare the relationship between realistic accumulation rate of antifouling biocides and response of biomarkers. Different concentrations of biocides and exposure time will also be important to test the modulatory effects.

In summary, a series of enzyme profiling with mRNA expression analysis can be a useful approach to understand the mode of action of emerging antifouling agents in oysters. Our results indicate that a gene or enzyme may respond to a certain antifouling agent via a specific response mechanism. The findings in the present study agree with the observations in mammals on antifouling biocide-triggered oxidative stress as well as cell stress response. Our data is also congruent with molecular and biochemical responses of mollusks associated with antifouling biocide contamination. Furthermore, enzyme profiling with mRNA expression analysis may shed light on the effects of unforeseen pollutants in marine ecosystems.

## Supporting Information

S1 Table Information of primer sets used in this study. (DOCX)
