# Supplementary material for: Effects of Antifouling Biocides on Molecular and Biochemical Defense System in the Gill of the Pacific Oyster Crassostrea gigas
Source: PLoS One. 2016 Dec 22;11(12):e0168978. doi: 10.1371/journal.pone.0168978 (PMC5179263; doi:10.1371/journal.pone.0168978)
Supplement: S1 Table — (DOCX) [file pone.0168978.s001.docx]

**S1 Table. Information of primer sets used in this study.** Detailed primer sets are retrieved from previous studies [1-4].

| **Gene** | **Oligo name** | **Sequence (5’→3’)** | **Amplicon length (bp)** |
| --- | --- | --- | --- |
| *EF1α* | RT-F | ACCACCCTGGTGAGATCAAG | 104 |
|  | RT-R | ACGACGATCGCATTTCTCTT |  |
| *GSTO* | RT-F | TGATGAGTTCACCACCGCAA | 155 |
|  | RT-R | TTCAAACCATGGCCACAGCA |  |
| *GSTP* | RT-F | CACCATTCACGACTTTGTGGCAGA | 185 |
|  | RT-R | TCAGCCATTTCGGTAGCCTCTCTT |  |
| *GSTS* | RT-F | AACGCCACCATTCACGAC | 118 |
|  | RT-R | AAGACCCCACCCAATGCT |  |
| *GPx* | RT-F | GGACTACCCGATGATGAACGA | 70 |
|  | RT-R | GGAAGGAAACCCGAGAACCA |  |
| *GR* | RT-F | TTCGCCCTGCTGCTATGG | 72 |
|  | RT-R | TTGCCCTGGGAGATGTTTG |  |
| *CuZnSOD* | RT-F | AACCCCTTCAACAAAGAGCA | 96 |
|  | RT-R | TTTGGCGACACCGTCTTC |  |
| *MnSOD* | RT-F | CATGTGCCAATCAAGATCCTC | 111 |
|  | RT-R | AGTCTGGTCGCACATTCTTGT |  |
| *Catalase* | RT-F | TTCGTCATATCGGGTTTACTTCTG | 75 |
|  | RT-R | CCTTGTCACGTCCTGCCATT |  |
| *ATPase-15152* | RT-F | ATGGAGCGTGAGAGGGACAAGG | 118 |
|  | RT-R | CTGAGCGGCGAGCAAACTTCAT |  |
| *AChE* | RT-F | GCAAGAAGATGATGAAGTATTGG | 88 |
|  | RT-R | CAGTCATCCAGACCTCTCTCTTC |  |
| *HSP70-02823* | RT-F | GCTGTGGCTTATGGAGCTGCTG | 105 |
|  | RT-R | TCCTGCCGTTTCAATGCCCAAA |  |
| *HSP70-02594* | RT-F | TCAGCCAAGGACAAGAGCACAG | 104 |
|  | RT-R | TCGGCCTCGTTCACCATTCTCT |  |
| *HSP70-08834* | RT-F | CCAGAACGACAACAACAGACTCTCA | 108 |
|  | RT-R | TTGGCTTCAACCTTCTCCTTCACTT |  |
| *HSP70-16262* | RT-F | TCACTCCGCTGTCGTTGGGTAT | 118 |
|  | RT-R | TGTCCATCAGCAGCCGTTGAGA |  |
| *HSP70-27222* | RT-F | TCCATACCAAGCCTGAGCTGAAGA | 80 |
|  | RT-R | TACACGGAGTAGCGACACATCCA |  |
| *HSP70-17255* | RT-F | ACCAAGCACAACTCTGAAT | 115 |
|  | RT-R | AGGCTGAGTATCCACAATG |  |
| *HSP70-13249* | RT-F | ATAGCCAGGGTTGGAGCAGGTT | 122 |
|  | RT-R | ACGGATACCGAGGTCAGCACTG |  |
| *HSP70-12492* | RT-F | CTGAAGATCCAGGACTTGCTGTGTT | 108 |
|  | RT-R | GCCAGCTCTGAATGCCATAAGTGT |  |
| *HSP70-02491* | RT-F | CCACGTCATCAGGTCGATACAGAGA | 111 |
|  | RT-R | GCAATATGCTGTCTACGGCTGGTT |  |
| *HSP90-17621* | RT-F | CGAGGAACAGAAGGCTGAGTACGA | 107 |
|  | RT-R | AAGGAGATGTCACCAGACGGTTAGA |  |
| *HSP90-25730* | RT-F | GGCAAGGACGACTACGAGAAGTTC | 130 |
|  | RT-R | CCGTGTCAGAGTTGGAGGAGTAGAA |  |
| *HSP60-18096* | RT-F | GCGTCCGGTGATCTACTGGTCT | 110 |
|  | RT-R | TGACACTGCGAGTCGTACTGGA |  |
| *HSP60-02375* | RT-F | TTGCTGACTCGGGCTGTACTGT | 103 |
|  | RT-R | GACATCAGGCGGACCACTAGGA |  |
| *HSP40-06977* | RT-F | CACATTTCCAGAAGAAGGCGACCA | 128 |
|  | RT-R | GAACCTTGGCAGTGTGGATCAGATT |  |
| *HSP40-09495* | RT-F | GGAGGAGACGACCCGTTTGCTA | 103 |
|  | RT-R | GTTGCCCGCCGAAATGGAACA |  |
| *HSP20-17582* | RT-F | CCGAAGGAAGAGGACCAGGAGATG | 133 |
|  | RT-R | CGAACACCGACAGGTCTAAACTCTC |  |
| *HSP20-04164* | RT-F | CGCCATTACGGACGGCAAGAA | 105 |
|  | RT-R | ACGGTAATGTGGTCAGGCTCGA |  |
| *HSF-07406* | RT-F | CCGACTCGTCAACATCATCAGACC | 107 |
|  | RT-R | TGCGTTTGTGGGCTTGGTATTCA |  |

**References**

1. Farcy É, Voiseux C, Lebel J-M, Fiévet B. Transcriptional expression levels of cell stress marker genes in the Pacific oyster *Crassostrea gigas* exposed to acute thermal stress. Cell Stress Chaperones. 2009;14(4):371-80. doi: 10.1007/s12192-008-0091-8. PubMed PMID: PMC2728272.

2. Béguel J-P, Huvet A, Quillien V, Lambert C, Fabioux C. Study of the antioxidant capacity in gills of the Pacific oyster Crassostrea gigas in link with its reproductive investment. Comp Biochem Physiol C Toxicol Pharmacol. 2013;157(1):63-71. doi: http://dx.doi.org/10.1016/j.cbpc.2012.10.004.

3. Serrano MAS, Gonzalez-Rey M, Mattos JJ, Flores-Nunes F, Mello ÁCP, Zacchi FL, et al. Differential gene transcription, biochemical responses, and cytotoxicity assessment in Pacific oyster Crassostrea gigas exposed to ibuprofen. Environ Sci Pollut Res Int. 2015;22(22):17375-85. doi: 10.1007/s11356-014-4023-0.

4. Zhu Q, Zhang L, Li L, Que H, Zhang G. Expression Characterization of Stress Genes Under High and Low Temperature Stresses in the Pacific Oyster, *Crassostrea gigas*. Mar Biotechnol. 2016;18(2):176-88. doi: 10.1007/s10126-015-9678-0.
